# Prevalence of Diagnosed Diabetes in Adults by Diabetes Type — United States, 2016

**DOI:** 10.15585/mmwr.mm6712a2

**Published:** 2018-03-30

**Authors:** Kai McKeever Bullard, Catherine C. Cowie, Sarah E. Lessem, Sharon H. Saydah, Andy Menke, Linda S. Geiss, Trevor J. Orchard, Deborah B. Rolka, Giuseppina Imperatore

**Affiliations:** ^1^Division of Diabetes Translation, National Center for Chronic Disease Prevention and Health Promotion, CDC; ^2^National Institute of Diabetes and Digestive and Kidney Diseases, National Institutes of Health, Bethesda, Maryland; ^3^National Center for Health Statistics, CDC; ^4^Social & Scientific Systems, Inc., Silver Spring, Maryland; ^5^Department of Epidemiology, Graduate School of Public Health, University of Pittsburgh, Pittsburgh, Pennsylvania.

Currently 23 million U.S. adults have been diagnosed with diabetes (*1*). The two most common forms of diabetes are type 1 and type 2. Type 1 diabetes results from the autoimmune destruction of the pancreas’s beta cells, which produce insulin. Persons with type 1 diabetes require insulin for survival; insulin may be given as a daily shot or continuously with an insulin pump (*2*). Type 2 diabetes is mainly caused by a combination of insulin resistance and relative insulin deficiency (*3*). A small proportion of diabetes cases might be types other than type 1 or type 2, such as maturity-onset diabetes of the young or latent autoimmune diabetes in adults (*3*). Although the majority of prevalent cases of type 1 and type 2 diabetes are in adults, national data on the prevalence of type 1 and type 2 in the U.S. adult population are sparse, in part because of the previous difficulty in classifying diabetes by type in surveys (*2*,*4*,*5*). In 2016, supplemental questions to help distinguish diabetes type were added to the National Health Interview Survey (NHIS) (*6*). This study used NHIS data from 2016 to estimate the prevalence of diagnosed diabetes among adults by primary type. Overall, based on self-reported type and current insulin use, 0.55% of U.S. adults had diagnosed type 1 diabetes, representing 1.3 million adults; 8.6% had diagnosed type 2 diabetes, representing 21.0 million adults. Of all diagnosed cases, 5.8% were type 1 diabetes, and 90.9% were type 2 diabetes; the remaining 3.3% of cases were other types of diabetes. Understanding the prevalence of diagnosed diabetes by type is important for monitoring trends, planning public health responses, assessing the burden of disease for education and management programs, and prioritizing national plans for future type-specific health services.

NHIS is an annual, cross-sectional household interview survey conducted by CDC that gathers health-related data in a nationally representative sample of the civilian, noninstitutionalized U.S. population (*6*). The 2016 NHIS Sample Adult Core consisted of 33,028 adults aged ≥18 years, with a final response rate of 54.3%. Each respondent was randomly selected among all adults aged ≥18 years in each household. During face-to-face interviews, respondents were asked whether a doctor or health care professional had ever told them that they had diabetes, other than during pregnancy. Among those who said they had diabetes, questions were asked regarding age at diagnosis and insulin and oral hypoglycemic medication use. In 2016, respondents were also asked to report whether they had type 1, type 2, or another type of diabetes. Virtually all patients with type 1 diabetes require insulin to survive, and very few persons who use insulin do not report using it (*5*). Previous studies have found that self-reported diabetes type alone is not a valid method for classifying diabetes type in surveys because some patients are not aware of their diabetes type (*5*,*7*). Therefore, for this analysis, type 1 diabetes was defined as current insulin use and self-report of type 1 diabetes. Adults who reported having type 1 diabetes but reported not using insulin were classified as having type 2 diabetes, as were persons who reported type 2 diabetes, unknown diabetes type, or who would not report diabetes type. Respondents who reported having another diabetes type were classified as having “other type.”

Crude prevalence estimates of diagnosed diabetes by type and 95% confidence intervals (CIs) were calculated for the overall population and by selected sociodemographic characteristics. P values were calculated from chi-squared tests and were considered significant at <0.05. Final survey weights were applied to the data to adjust for various probabilities of selection and household nonresponse. Statistical software was used to account for NHIS’s complex sampling design.

A total of 3,519 respondents aged ≥18 years reported having diabetes, including 211 classified as having type 1; 3,210 classified as having type 2 (including 182 who reported having type 1, but not taking insulin; 2,897 who reported having type 2; one who reported an unknown type; and one refusal); and 98 classified as having “other” type. In 2016, the overall crude prevalence of diagnosed diabetes among U.S. adults was 9.44% (95% CI = 9.01–9.88). The prevalences of type 1 diabetes, type 2 diabetes, and other diabetes types were 0.55%, 8.58%, and 0.31%, respectively (Table). The weighted percentages of all diagnosed diabetes cases that were type 1 and type 2 were 5.8% and 90.9%, respectively; the remaining were other types. Based on the weighted NHIS population, the estimated numbers of adults with type 1, type 2, and other diabetes types were 1.3 million, 21.0 million, and 0.8 million, respectively.

**TABLE:** Crude prevalence* of diagnosed diabetes among adults by diabetes type^†^ and selected characteristics — National Health Interview Survey, United States, 2016

Characteristic	Diabetes type		
	Type 1	Type 2	Other type
	% (95% CI)^§^	% (95% CI)^§^	% (95% CI)^§^
**Total**	**0.55 (0.46–0.66)**	**8.58 (8.17–9.00)**	**0.31 (0.24–0.40)**
**Age group (yrs)**			
18–29	0.45 (0.27–0.75)	0.66 (0.38–1.13)	—^¶^
30–44	0.50 (0.35–0.73)	3.29 (2.75–3.93)	0.27 (0.16–0.47)
45–64	0.59 (0.44–0.78)	11.03 (10.24–11.88)	0.44 (0.30–0.65)
≥65	0.65 (0.48–0.88)	19.62 (18.54–20.74)	0.35 (0.23–0.54)
**Sex**			
Men	0.64 (0.51–0.82)	8.86 (8.30–9.45)	0.23 (0.15–0.36)
Women	0.46 (0.37–0.58)	8.32 (7.79–8.88)	0.38 (0.27–0.53)
**Race/Ethnicity**			
White, non-Hispanic	0.67 (0.55–0.82)	7.99 (7.54–8.45)	0.29 (0.21–0.39)
Black, non-Hispanic	0.45 (0.26–0.78)	11.52 (10.35–12.80)	0.45 (0.24–0.86)
Asian, non-Hispanic	—^¶^	6.89 (5.24–9.03)	—^¶^
Hispanic	0.22 (0.12–0.40)	9.07 (7.91–10.38)	—^¶^
**Education level**			
<High school	0.83 (0.56–1.24)	14.20 (12.88–15.64)	0.56 (0.32–0.98)
High school	0.58 (0.40–0.84)	9.99 (9.18–10.86)	0.29 (0.17–0.47)
>High school	0.48 (0.39–0.61)	6.89 (6.47–7.34)	0.27 (0.19–0.38)

**Abbreviation:** CI = confidence interval.

* Overall crude prevalence of diagnosed diabetes = 9.44% (95% CI = 9.01–9.88).

^†^ Type 1 diabetes was defined as self-report of type 1 diabetes and current insulin use. Respondents who self-reported other diabetes types were classified as having “other type” diabetes. All remaining cases were classified as type 2 diabetes.

^§^ Estimates are weighted percentages and 95% CIs. CIs were based on a logit transformation and might be asymmetric about the point estimate.

^¶^ Estimate might be unreliable because of large relative standard error (>30%); data not shown.

Estimated crude prevalence of type 1 diabetes among U.S. adults did not significantly vary by age group (p = 0.54) or education (p = 0.14) (Table). The prevalence of type 1 diabetes was higher among men (0.64%) than among women (0.46%) (p<0.05) and higher among non-Hispanic whites (whites) (0.67%) than among Hispanics (0.22%) (p<0.01). By age group, the prevalence of type 2 diabetes was highest among adults aged ≥65 years and lowest among adults aged 18–29 years (p<0.001), and by race/ethnicity, was higher among non-Hispanic blacks (11.52%) than among non-Hispanic Asians (6.89%), whites (7.99%), and Hispanics (9.07%) (p<0.001) (Table). The prevalence of type 2 diabetes decreased with higher levels of educational attainment (p<0.001).

## Discussion

In 2016, the estimated prevalences of diagnosed type 1 and type 2 diabetes were 0.55% (corresponding to 1.3 million U.S. adults) and 8.6% (corresponding to 21.0 million U.S. adults), respectively. Type 1 and type 2 diabetes accounted for approximately 6% and 91% of all cases of diagnosed diabetes, respectively. Because the prevalence of type 2 diabetes is so much higher than that of type 1, current diabetes surveillance data that do not distinguish diabetes type are more reflective of persons with type 2 diabetes. Recent analysis of diagnosed diabetes prevalence indicates a plateauing among adults aged 20–79 years (*8*), but it is not known whether this trend might differ for type 1 diabetes. Because the etiology, treatment, and outcomes of diabetes vary by type, it is important to distinguish between them.

There is no reference standard for classifying prevalent type 1 diabetes or type 2 diabetes cases in public health surveillance. The presence of autoantibodies against the beta cells of the pancreas and the lack of endogenous insulin secretion are biologic markers of type 1 diabetes. However, beta cell autoantibodies disappear with time and might even be absent at the time of type 1 diabetes diagnosis (*2*). Insulin secretion tests are difficult to perform and interpret, making these tests unsuitable for use in cross-sectional surveys. In administrative health databases and electronic medical records, adults with diabetes frequently have *International Classification of Diseases* codes for both type 1 and type 2 diabetes. For this reason, disease coding has been combined with other information (e.g., current prescriptions for insulin or oral hypoglycemic medication) when estimating diabetes type in these data (*9*,*10*). Using type 1 diabetes self-report and current insulin use to classify diabetes type, the percentage of all diabetes cases that were type 1 diabetes fell reasonably within the range of results from other studies (approximately 5%–10%) (*3*–*5*,*9*).

The findings in this report are subject to at least three limitations. First, the data were self-reported and underestimate the total number of adults with diabetes. Second, data were not validated, which could have led to misclassification of diabetes type. Adults with self-reported type 1 diabetes who did not report insulin use were reclassified as having type 2 diabetes, which might have resulted in misclassification if they actually used insulin but did not report use. However, self-reported use of insulin is highly specific: <0.02% of persons who reported insulin in a medication log failed to report using it when asked (*5*). Some insulin users with type 2 diabetes might have incorrectly reported type 1 diabetes, assuming that taking insulin meant they had type 1 diabetes (*5*). In addition, because self-reported cases of unknown type were reclassified as type 2 diabetes, the prevalence of type 2 diabetes might have been overestimated. However, according to a Canadian survey-based algorithm to distinguish diabetes types, 99% of adults who self-reported unknown type would have been classified as type 2 diabetes (*7*). Finally, the small sample size of some subgroups limited precision.

Despite these limitations, this first study to estimate the prevalence of diagnosed type 1 and type 2 diabetes based on self-report and current insulin use among U.S. adults provides information to track prevalence of diabetes by type to monitor trends and assess the burden of disease for education and prevention programs. Knowledge about national prevalences of type 1 and type 2 diabetes might facilitate assessment of the long-term cost-effectiveness of public health interventions and policies aimed at improving diabetes management and help to prioritize national plans for future type-specific health services.

**Summary**

**What is already known about this topic?**

The two most common forms of diabetes are type 1 and type 2. Previous national diabetes prevalence estimates did not distinguish between types among U.S. adults.

**What is added by this report?**

New data allowed estimation of diagnosed diabetes by type. In 2016, the prevalence of diagnosed type 1 diabetes was 0.55%, representing 1.3 million U.S. adults; the prevalence of diagnosed type 2 diabetes was 8.6%, representing 21.0 million U.S. adults. Non-Hispanic white adults had a higher prevalence of diagnosed type 1 diabetes than did Hispanic adults. Non-Hispanic blacks had the highest prevalence of diagnosed type 2 diabetes. Diagnosed type 2 diabetes prevalence estimates increased with age and decreased with increasing levels of educational attainment.

**What are the implications for public health practice?**

Knowledge about national prevalence of diagnosed diabetes by type might be helpful in monitoring trends, assessing the burden of disease for education and management programs, and guiding and prioritizing national plans for future type-specific health services.
